# Study on the Mechanism of miR-125b-5p Affecting Melanocyte Biological Behavior and Melanogenesis in Vitiligo through Regulation of MITF

**DOI:** 10.1155/2022/6832680

**Published:** 2022-11-16

**Authors:** Xiaochuan Wang, Yifei Wu, Peng Du, Liangheng Xu, Yuan Chen, Jingzi Li, Xuying Pan, Zhiqiong Wang

**Affiliations:** Department of Dermatology, The First People's Hospital of Yunnan Province, Kunming, 650032 Yunnan, China

## Abstract

**Objective:**

The goal was to confirm the mechanism by which miR-125b-5p influences melanocyte biological behavior and melanogenesis in vitiligo by regulating MITF.

**Methods:**

oe-MITF, sh-MITF, miR-125b-5p mimic, NC-mimic, NC-inhibitor, and miR-125b-5p inhibitor were transfected into cells by cell transfection. Western blotting was used to detect the related protein expression, qRT–PCR was used to detect miR-125b-5p and MITF expression, immunohistochemistry was used to detect the MITF-positive cells in vitiligo patients tissues, and a dual-luciferase reporter system was used to detect the target of miR-125b-5p and MITF. PIG1 and PIG3V cell proliferation by the CCK-8 method, cell cycle progression and apoptosis by flow cytometry, apoptosis was detected by TUNEL, Tyr activity and melanin content were measured using Tyr and melanin content assay kits.

**Results:**

Compared with the healthy control group, the expression of miR-125b-5p in the tissues and serum of vitiligo patients was upregulated, and the expression of MITF was downregulated; compared with PIG1 cells, the expression of miR-125b-5p and MITF in the PIG3V group was consistent with the above. Compared with the NC-minic group, the cell proliferation activity of the miR-125b-5p mimic group decreased, apoptosis increased, and the expression levels of melanogenesis-related proteins Tyr, Tyrp1, Tyrp2, and DCT were downregulated. Compared with the NC-inhibitor group, the above indices in the miR-125b-5p inhibitor group were all opposite to those in the miR-125b-5p mimic group. Transfection of oe-MITF into the miR-125b-5p mimic group reversed the effect of the miR-125b-5p mimic, while transfection of sh-MITF enhanced the effect of the miR-125b-5p mimic.

**Conclusion:**

miR-125b-5p affects vitiligo melanocyte biological behavior and melanogenesis by downregulating MITF expression.

## 1. Introduction

Vitiligo characterized by melanocyte destruction is an acquired and idiopathic disease resulting in dilution of pigment in affected areas [1]. Its lesion sites are characterized by white patches and colorless, nonscaly, and distinct edges. With research advances, great progress has been made in the understanding of the pathogenesis of vitiligo, which is associated with the destruction of melanocyte function leading to impaired melanin deposition as an autoimmune disease [2]. Nonetheless, the mechanism of melanin deposition disorder is not clear in the current study; therefore, there is an urgent need to study the specific mechanism of melanin deposition and find effective therapeutic targets.

As an evolutionarily conserved noncoding RNA molecule, microRNA (miRNA) is approximately 22 nucleotides. miRNA bind to their target mRNA to regulate gene expression, resulting in their translation inhibition or degradation. Each miRNA has multiple targets, and each miRNA is regulated by multiple miRNAs simultaneously [3]. Previous studies have shown that miRNAs play a key role in all kinds of life activities, including the immune response, cell proliferation, differentiation, and apoptosis [4]. There are four kinds of miRNAs with abnormal expression in the peripheral blood of vitiligo patients: miR-1238-3p, miR-202-3p, miR-630, and miR-766-3p [5]. Upregulation of miR-21-5p and downregulation of SOX5 lead to upregulation of melanogenesis [6]. miR-211 can regulate mitochondrial energy metabolism in vitiligo patients [7]. The relationship between miR-125b-5p, melanocyte biological behavior, and melanogenesis was investigated. As a member of the miR-125b family, miR-125b-5p is a new regulator of homeostatic melanogenesis [8]. However, the specific mechanism by which miR-125b-5p regulates melanocyte biological behavior and melanogenesis is not clear.

As a key regulator of melanocyte development, MITF (small eye transcription factor) is necessary in many stages of the melanocyte life cycle and is required for the survival of melanocyte precursors or melanoblasts [9]. In a study by Steingrímsson et al. [10], deletion of MITF resulted in the entire absence of mouse embryonic adult melanocytes, and mice were born with all-white hair follicles. Biological information indicates that miR-125b-5p interacts with MITF, but whether miR-125b-5p influences the biological behavior of melanocytes and melanin production by regulating MITF is not known. Therefore, this study was aimed at confirming the relationship between miR-125b-5p, MITF, melanocyte biological behavior, and melanogenesis to explore the potential mechanism and provide a new way to treat vitiligo.

## 2. Methods

### 2.1. Clinical Data and Patient Samples

In this study, serum and skin tissue were collected from patients with vitiligo (*n* = 17) and normal healthy individuals (*n* = 17). Patients who had other serious diseases or started treatment within 3 months prior to admission were excluded. Healthy controls were shown to have normal physiological functions in a whole-body physiological examination performed at the abovementioned hospital. All the experiments in this study were approved by the ethics committee. Before participating in this study, the included patients and healthy volunteers signed informed consent forms.

### 2.2. Cell Culture

Normal human skin melanocytes (PIG1) and vitiligo melanocytes (PIG3V) (BeNa Culture Collection, Beijing, China) were cultured in 90% RPMI1640 containing fetal bovine serum (10% USA), penicillin (100 U/mL), and streptomycin (100 mg/mL). The cells were placed in a standard incubator with 5% CO_2_ and incubated at a cell density of 80% for subsequent experiments.

### 2.3. Cell Transfection

In 6-well plates, PIG1 and PIG3V cells were inoculated at a density of 1 × 10^5^ cells/well. miR-125b-5p mimic, sh-MITF, oe-MITF, NC-mimic, NC-inhibitor, and miR-125b-5p inhibitor were synthesized by Sangon (Shanghai, China) and transfected into cells by Lipofectamine™ 3000 reagent. The control group was transfected into cells as a control group, and the cells were subsequently placed in saturated humidity and CO_2_ for 48 h. The transfection efficiency was detected using qRT–PCR and WB for subsequent experiments.

### 2.4. qRT–PCR

A total RNA extractor (Sangon Biotech) was used to extract the total RNA from 17 serum samples (tissues and cells). A 1 *μ*L RNA sample was taken, and RNA integrity was detected by 1% agarose gel electrophoresis. A 1 *μ*L RNA sample was taken after dilution to measure the OD value through the ratio of OD_260_/OD_280_ to identify the total RNA purity. A cDNA synthesis kit (Vazyme, Nanjing, China) was used to reverse transcribe 2 *μ*g of mRNA into cDNA, which was then diluted 10 times. One microliter of the prepared cDNA was used for qPCR. U6 and GAPDH were used as the reference genes for expression detection on an ABI7500 real-time PCR system, followed by PCR analysis of cDNA using SYBR qPCR Master Mix (Vazyme, Nanjing, China) to quantify miR-125b-5p and MITF expression. qRT–PCR conditions are as follows: 95°C for 30 s, 3 s at 95°C, followed by annealing at 60°C for 30 s for 40 cycles. All primers (Table 1) used in this study were designed with Premier 5.0. The results were calculated by the 2-*ΔΔ*Ct method after repetition at least 3 times.

### 2.5. Cell Proliferation Assay (CCK-8)

In this study, PIG1 and PIG3V cells (1 × 10^5^ cells/well) were placed in 96-well plates. In a 37°C incubator, 100 *μ*L of medium was added to every plate and cultured in a cell incubator for 36 h until the cell density was 70-80%. After transfection or dosing and incubation for 0, 24, 48, and 72 h, ten microliters of CCK-8 reagent (Biotrans, Shanghai, China) was added to detect the influence of miR-125b-5p and MITF on cells. Subsequently, the 96-well plates were subjected to an enzyme marker (Bio-Rad, CA, USA) at 450 nm to detect the absorbance values.

### 2.6. Cell Clone Formation Experiments

To prepare the cell suspensions, we used trypsin (0.25%) to digest the PIG1 and PIG3V cells at the logarithmic growth stage. After gradient dilution, the cell suspension was inoculated into a 37°C plate containing 10 mL of preheated culture medium at gradient densities of 50, 100, and 200 cells. Then, the plate was gently rotated to evenly distribute the cells, and the cells were cultured in a cell incubator. They were observed frequently, and when clones visible to the naked eye appeared in the dishes, the culture was terminated, the supernatant was discarded, the cells were washed twice with PBS, 15 mL of 4% paraformaldehyde was added to fix the cells, the fixation was discarded, GIMSA application staining solution (Jinglai Biological, China) was added to the dye for 10-30 min, and they were then washed with water, air-dried, and counted under a microscope.

### 2.7. Protein Blotting

In this study, the proteins were extracted utilizing RIPA lysis buffer (Sangon Biotech, Shanghai), and a lysate containing benzoyl fluoride (PMSF) was added. A BCA assay (Sangon Biotech, Shanghai) was used to determine the total protein content. The target bands were transferred to a nitrocellulose membrane (PVDF) by taking 50 *μ*g for 10% SDS–PAGE and using skim milk powder (5%) to block the PVDF membrane for 2 h. PVDF membranes were cultured with Abcam antibodies: MITF (1/1000; cat. no. ab140606, Abcam, UK), CDK2 (1 : 5000; cat. no. ab32147, Abcam, UK), CDK4 (1/1000; cat. no. ab108357, Abcam, UK), CyclinA2 (1/20000; cat. no. ab181591, Abcam, UK), CyclinD1 (1/200; cat. no. ab16663, Abcam, UK), BcL-2 (1/1000; cat. no. ab32124, Abcam, UK), Bax (1/1000; cat. no. ab32503, Abcam, UK), Caspase-3 (1/5000; cat. no. ab32351, Abcam, UK), Tyr (1/100000; cat. no. ab137869, Abcam, UK), Tyrp1 (1/1000; cat. no. ab235447, Abcam, UK), Tyrp2 (1/1000; cat. no. ab221144, Abcam, UK), DCT (1/1000; cat. no. ab221144, Abcam, UK), and GAPDH (1/1000; cat. no. ab9485, Abcam, UK) overnight at 4°C. TBST buffer was used to wash the PVDF membranes, which were then incubated with secondary antibodies (1/2000, cat. no. ab205718, Abcam) at 25°C for 1 h. Subsequently, ECL color development, gel imaging system analysis, semiquantitative determination of expression, and ImageJ analysis of the grayscale values of the bands were performed.

### 2.8. Immunohistochemistry (IHC)

IHC experiments were carried out by 3,3′-diaminobenzidine (DAB) analysis. First, paraffin sections were routinely dewaxed and incubated with 3% H_2_O_2_ for 10 min at 37°C (to inactivate endogenous peroxides), rinsed with distilled water, immersed in citrate buffer in a boiling water bath for antigen repair, rinsed, and then immersed three times with PBS for 5 min each. After the glass slide was baked at 65°C for 2 h, it was placed in xylene for 10 min and then treated with xylene for 10 min. The sections were incubated in the following ethanol gradient (5 min for each solution): 90%, 80%, 70%, and distilled water. In a wet room, citric acid buffer was used to treat the slices, and hydrogen peroxide (3%) was used to remove endogenous peroxidase (25°C, 10 min). Sections were blocked with 5% bovine serum at 37°C for 30 min and then incubated with the primary antibody for 12 h at 4°C. They were incubated with the second antibody for 30 min at 37°C after washing the slices with PBS buffer. 3,3′-Diaminobenzidine (DAB) was used to observe the sections, and a light microscope was used to acquire the images.

### 2.9. Flow Cytometry Detects the Cell Cycle and Apoptosis

PIG1 and PIG3V cells at the logarithmic growth stage from each group of treatments were inoculated in 6 cm culture dishes and cultured for 12 h. Subsequently, PBS was used to wash the cells three times, and the cells were resuspended in 100 *μ*L of buffer. At 25°C, the cells were coincubated with 5 *μ*L of PI (BD Biosciences) for 10 min. Finally, after adding termination buffer, flow cytometry was performed to determine the apoptosis rate. Images were processed and analyzed using FlowJo X software. The experiment was performed 3 times independently.

### 2.10. Dual-Luciferase Reporter Assay

Dual-luciferase reporter analysis was used to detect the relationship between miR-125b-5p and MITF. The StarBase (http://starbase.sysu.edu.cn/) database was used to confirm the targeting of miR-125b-5p with MITF. The 3′-UTR of MITF was ligated into the pmir-GLO vector to construct the MITF wild-type vector (MITF-WT). In addition, it was ligated into the pmir-GLO vector by changing the target binding site of miR-125b-5p and MITF using a gene mutation technique to construct an MITF mutant vector (MITF-MUT). With the miR-125b-5p mimic, the generated reporter plasmid was transfected into HEK293T cells, and a dual-luciferase reporter assay kit was used to measure the luciferase activity after 48 h of incubation.

### 2.11. Terminal deoxynucleotidyl transferase dUTP nick end labeling (TUNEL) Assay

According to the TUNEL kit (Beyotime, Shanghai, China), PIG1 and PIG3V cells at the logarithmic growth stage were taken, prepared in cell suspension by trypsin digestion, centrifuged, supernatant discarded, washed with PBS, and fixed with 4% paraformaldehyde solution at 25°C for 30 min. Subsequently, a 0.3% H_2_O_2_ methanol solution was used to block the cells. Of note, care should be taken to keep the surrounding area moist with sufficient water-soaked paper or cotton pads to minimize evaporation of the TUNEL assay solution during incubation. The TUNEL assay solution was washed off with PBS. The slices were sealed with antifluorescence quenching blocking solution, and the tissue and cells were photographed under a fluorescence microscope at 450-500 nm (400857, Nikon, Japan). PI/DAPI can stain both apoptotic and nonapoptotic cells red/blue, with green fluorescence localized by FITC-12-dUTP doping only in the nuclei of apoptotic cells.

### 2.12. Statistics and Analysis

In this study, GraphPad Prism 8 software was used to analyze and prepare graphs, and the means and standard deviations (SD) are shown. Unpaired one-way analysis and Student's *t* test were used to analyze the multiple groups and two groups of data, respectively. The *P* value for statistical significance was 0.05.

## 3. Results

### 3.1. Differential Expression of miR-125b-5p and MITF in the Serum, Melanocytes, and Tissues of Vitiligo Patients

**Figure 1:**
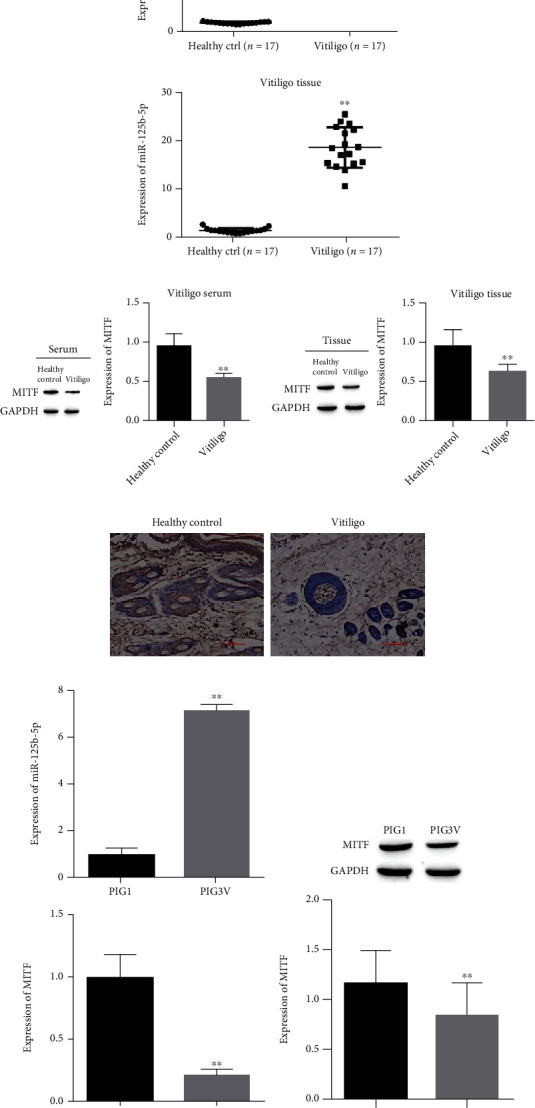
Differential levels of miR-125b-5p and MITF in the serum, tissues, and melanocytes of vitiligo patients. (a) RT-qPCR for differential expression of miR-125b-5p and MITF; (b) Western blot for MITF expression; (c) immunohistochemical detection of vitiligo patients' tissues; (d) RT–qPCR for differential levels of miR-125b-5p and MITF in PIG1 and PIG; (e) Western blot for differential levels of miR-125b-5p and MITF in PIG1 and PIG3V. ^∗∗^*P* < 0.01.

This study used qRT–PCR to confirm the levels of miR-125b-5p and MITF in serum and tissues from 17 vitiligo patients and 17 healthy individuals. It was shown that in either serum or tissues in the vitiligo patient group, miR-125b-5p expression was increased compared to the healthy controls (Figure 1(a)). Western blotting was used to detect MITF expression. MITF expression was significantly downregulated in vitiligo patients (Figure 1(b)). Immunohistochemical results indicated a significant decrease in MITF expressions from vitiligo patient tissues (Figure 1(c)). In melanocytes by qRT–PCR compared to normal human skin melanocytes (PIG1) and in vitiligo melanocytes (PIG3V), the level of miR-125b-5p was increased, and MITF was decreased (Figure 1(d)). At the same time, the level of MITF was significantly reduced in PIG3V cells according to Western blotting (Figure 1(e)). miR-125b-5p and MITF are abnormally expressed in vitiligo and may play a key role in the development of vitiligo.

### 3.2. The Influence of miR-125b-5p on Apoptosis, Melanogenesis, and the Proliferation of Melanocytes and Cycle and Pigment PIG1 and PIG3V Cells

**Figure 2:**
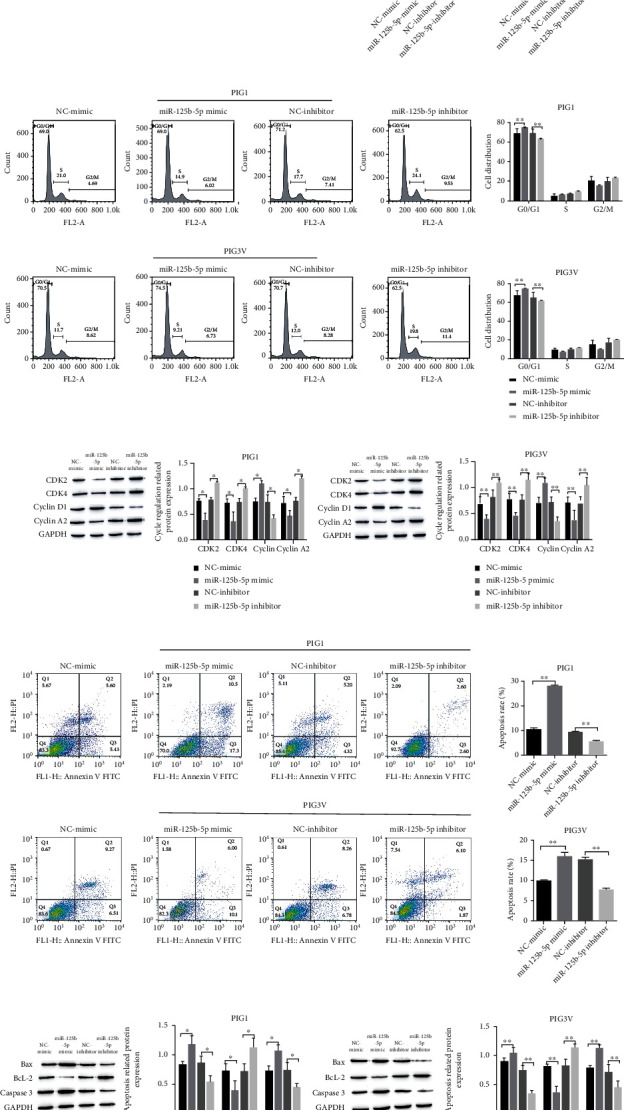
The influence of miR-125b-5p on the apoptosis, cycle, proliferation, and melanogenesis of melanocytes PIG1 and PIG3V. (a, b) CCK-8 and cell cloning experiments to evaluate the proliferation of PIG1 and PIG3V cells; (c, d) evaluation of the cell cycle of PIG1 and PIG3V by Western blot and flow cytometry; (e, f) detection of the apoptosis of PIG1 and PIG3V cells by Western blot and flow cytometry; (g) Western blot detection of melanogenesis-related protein expression. ^∗^*P* < 0.05; ^∗∗^*P* < 0.01.

To confirm the biological function of miR-125b-5p in vitiligo development, we altered the level of miR-125b-5p in cells by transfecting miR-125b-5p mimic and miR-125b-5p inhibitor. CCK-8 was used to evaluate the proliferation of PIG1 and PIG3V cells, as shown in Figure 2(a). Compared with the NC-minic group, the cell proliferation activity of the miR-125b-5p mimic group decreased, while the cell proliferation activity of the NC-inhibitor group showed no significant change. However, compared with the NC-inhibitor group, the cell proliferation activity of the miR-125b-5p inhibitor group was significantly increased. In addition, the influence of miR-125b-5p on PIG3V cells was found to be stronger than that on PIG1 cells during the assay. The cell clone formation assay was performed again, and the assay results were consistent with the CCK-8 results, as shown in Figure 2(b).

The cell cycle and apoptosis were detected by flow cytometry, and the miR-125b-5p mimic group showed G0/G1 phase; the levels of cycle regulation-related proteins CDK2, CDK4, and CyclinA2 protein were decreased, and CyclinD1 expression was increased. Meanwhile, apoptosis was increased and apoptosis-related protein BcL-2 expression was decreased, the expressions of Bax and Caspase-3 were increased, and the above indicators in the NC-inhibitor group did not change significantly. However, compared with the NC-inhibitor group, the changing trend of the miR-125b-5p inhibitor group was completely opposite to that of the miR-125b-5p group. The duration of the G0/G1 and S phase was reduced, the levels of cycle regulation-related CDK2, CDK4, and CyclinA2 proteins were increased, and CyclinD1 expression was decreased; meanwhile, apoptosis was attenuated, the BcL-2 level was increased, and Bax and Caspase-3 expressions were decreased (Figures 2(c)–2(f)).

The levels of melanogenesis-related proteins were detected by Western blotting. Tyr, Tyrp1, Tyrp2, and DCT were downregulated after treatment with the miR-125b-5p mimic, while all of them were upregulated in the miR-125b-5p inhibitor group (Figure 2(g)).

### 3.3. miR-125b-5p Targets and Negatively Regulates MITF Expression

**Figure 3:**
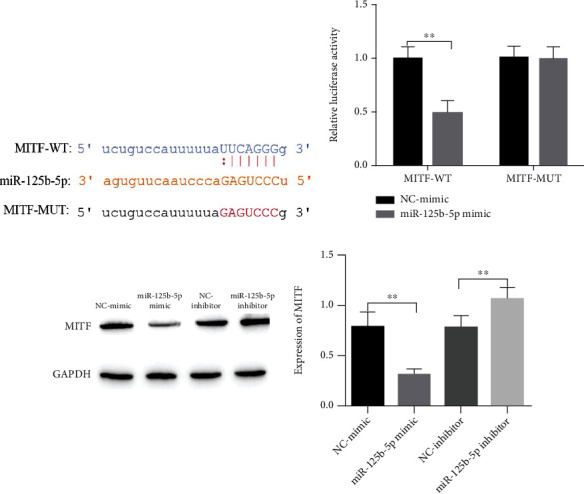
miR-125b-5p targets and negatively regulates MITF expression. (a) miR-125b-5p and MITF binding sequence; (b) dual-luciferase detection of miR-125b-5p in regard to MITF targeting; (c) Western blot detection of miR-125b-5p in regard to MITF regulation, ^∗∗^*P* < 0.01.

miRNAs can specifically bind to the 3′-untranslated region (3′-UTR) of target mRNAs to regulate gene expression. Therefore, we used a bioinformatics database to identify the target sites of miR-125b-5p with MITF and showed that there are MITF binding sites in miR-125b-5p (Figure 3(a)). Subsequently, a dual-luciferase reporter was used to analyze the miR-125b-5p and MITF targeting relationship. Overexpression of miR-125b-5p inhibited theluciferase activity of MITF-WT, while it had no significant effect on the luciferase activity of MITF-MUT (Figure 3(b)). Western blotting was used to examine the regulatory relationship between miR-125b-5p and MITF, and the results showed that the miR-125b-5p mimic decreased the levels of MITF, while the inhibitor increased the level of MITF (Figure 3(c)).

### 3.4. miR-125b-5p Affects PIG1 and PIG3V Proliferation and Apoptosis by Regulating MITF

**Figure 4:**
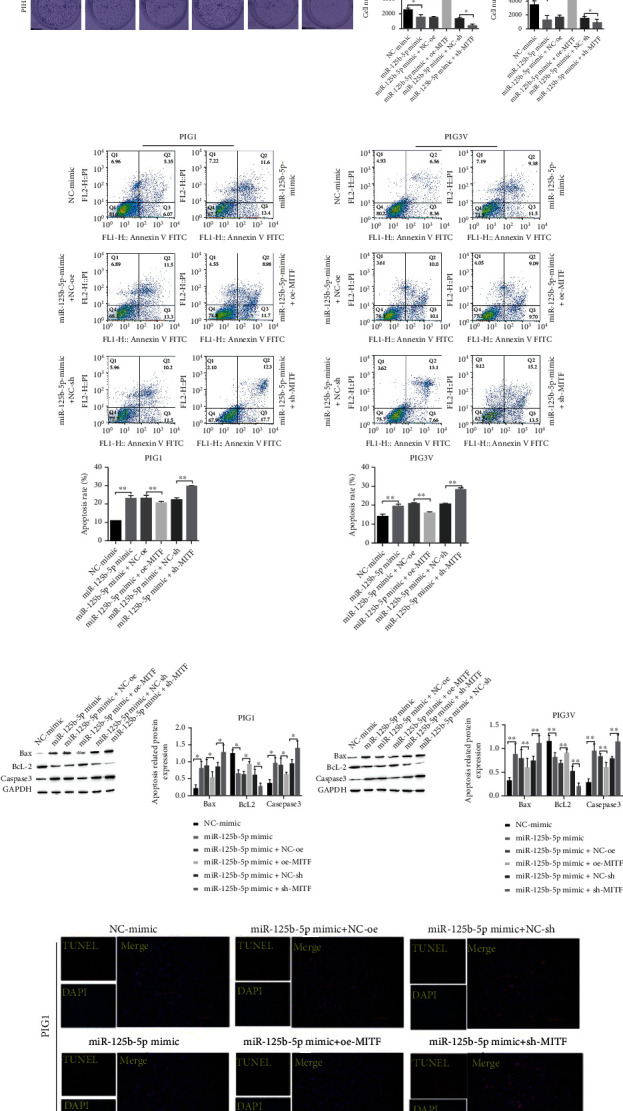
miR-125b-5p influences PIG1 and PIG3V apoptosis and proliferation through targeted regulation of MITF. (a, b) CCK-8 and cell cloning experiments to evaluate the proliferation of PIG1 and PIG3V cells, ^∗∗^*P* < 0.01; (c, d) PIG1 and PIG3V apoptosis, ^∗^*P* < 0.05 and ^∗∗^*P* < 0.01; (e) detection of PIG1 and PIG3V cell apoptosis by TUNEL.

To confirm whether miR-125b-5p affects PIG1 and PIG3V proliferation and apoptosis by targeting regulating MITF, miR-125b-5p with MITF overexpression and a low expression vector were constructed to alter the level of miR-125b-5p with MITF. A CCK-8 assay was used to examine the proliferation of cells after 72 h of growth. Compared with the NC-mimic group, the proliferation activity of the miR-125b-5p mimic group was decreased. After cotransfection of oe-MITF with miR-125b-5p mimic, compared with the miR-125b-5p mimic+NE-oe group, the miR-125b-5p mimic+oe-MITF group can effectively reverse the inhibitory effect of miR-125b-5p mimic on cell proliferation. After the expression of MITF was inhibited, the inhibitory effect of miR-125b-5p mimic on the proliferation of PIG1 and PIG3V cells was further enhanced (Figure 4(a)). The results of the cell clone formation assay were consistent with those of the CCK-8 assay of cell proliferation (Figure 4(b)).

Flow cytometry was used to detect PIG1 and PIG3V apoptosis in this study. The level of apoptosis was increased under the treatment of miR-125b-5p mimic, Caspase-3 and Bax expression was increased, but BcL-2 protein was decreased. After overexpression of MITF, the effect of miR-125b-5p alone was reversed, and apoptosis was attenuated, but BcL-2 was upregulated, and Bax and Caspase-3 were downregulated. If the expression of MITF was decreased while overexpressing miR-125b-5p, it promoted the effect of overexpressing miR-125b-5p alone, apoptosis was increased again, and the expression of apoptosis-related protein BcL-2 was decreased again, but Bax and Caspase-3 were increased again (Figures 4(c) and 4(d)).

TUNEL assay of apoptosis in PIG1 and PIG3V cells showed an increase in apoptosis under the treatment of the miR-125b-5p mimic, cotransfection of oe-MITF with it partially reversed the influence of miR-125b-5p mimic transfection alone, and apoptosis was attenuated. However, cotransfection of sh-MITF with it promoted the effect of transfection of the miR-125b-5p mimic alone and apoptosis was diminished, while cotransfection of sh-MITF with it promoted the influence of the treatment of miR-125b-5p mimic alone, and apoptosis was increased again (Figure 4(e)). In summary, miR-125b-5p influences the proliferation and apoptosis of PIG1 and PIG3V cells by targeting the negative regulation of MITF expression and has a greater effect on PIG3V.

### 3.5. miR-125b-5p Affects the PIG1 and PIG3V Cycles by Targeting Regulating MITF

**Figure 5:**
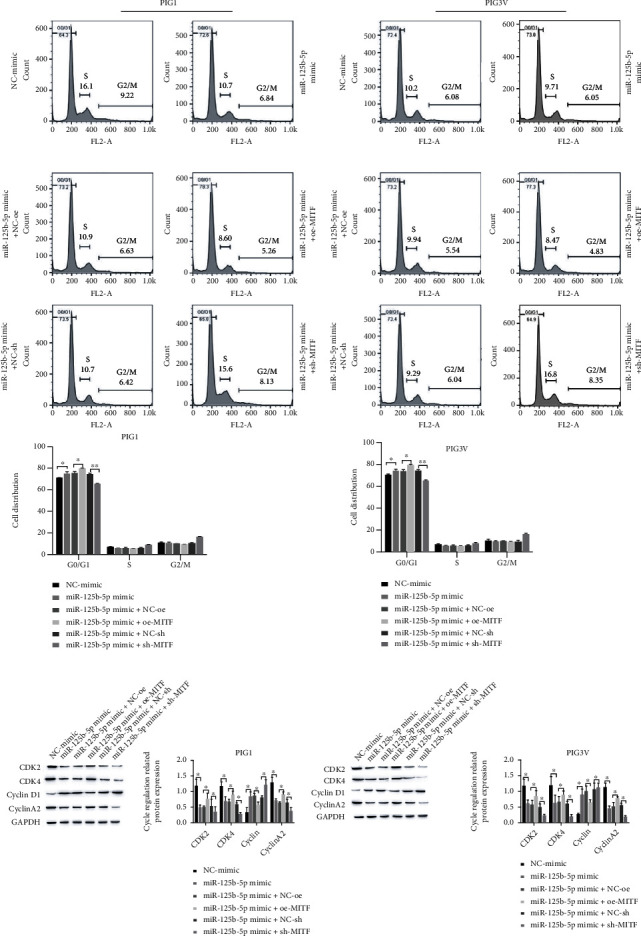
miR-125b-5p influences the cycle of PIG1 and PIG3V by targeting and regulating MITF. (a) Flow cytometry detection of cellular PIG1 and PIG3V cycles; (b) detection of cycle-regulated related proteins by Western blot, ^∗^*P* < 0.05 and ^∗∗^*P* < 0.01.

Using flow cytometry to detect cell PIG1 and PIG3V cycles, the results showed that, under the treatment of the miR-125b-5p mimic, cells had G0/G1 and S phase block; downregulated CDK2, CDK4, and CyclinA2 protein expression; and upregulated CyclinD1 expression. After cooverexpressing MITF, cell G0/G1 phase and S phase block was relieved; CDK2, CDK4, and CyclinA2 protein expressions were upregulated; and CyclinD1 expression was downregulated. However, when knocking down MITF, under the treatment of miR-125b-5p mimic+NE-sh, cells in the miR-125b-5p mimic+sh-MITF group had G0/G1 and S phase block; CDK2, CDK4, and CyclinA2 protein expressions were decreased; and CyclinD1 expression was increased (Figures 5(a) and 5(b)).

### 3.6. miR-125b-5p Affects Melanogenesis of PIG1 and PIG3V through Target Regulation of MITF

**Figure 6:**
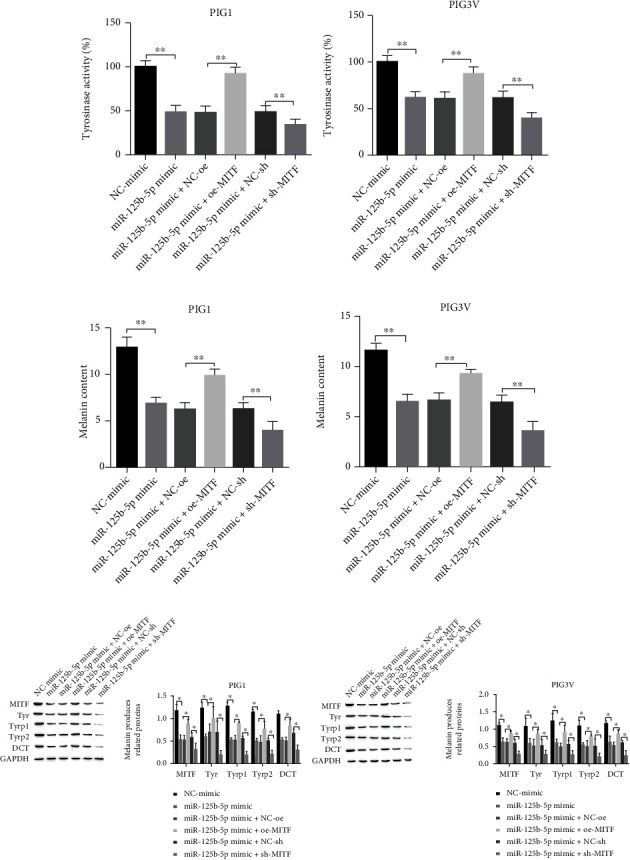
miR-125b-5p influences melanogenesis of PIG1 and PIG3V through target regulation of MITF. (a) Try activity assay; (b) melanin content measurement; (c) Western blot for the level of melanin production-related protein. ^∗^*P* < 0.05 and ^∗∗^*P* < 0.01.

To confirm whether miR-125b-5p can affect PIG1 and PIG3V melanin production by regulating MITF expression, we examined the activity of Tyr, the melanin content, and melanogenesis-related protein expression. The results showed that, compared with the miR-125b-5p mimic+NE-oe group, Tyr activity was enhanced under the treatment of with cotransfection of oe-MITF with the miR-125b-5p mimic. Tyr activity was reduced when MITF was knocked down because it reduced the inhibitory effect of the miR-125b-5p mimic on Tyr activity (Figure 6(a)).

Melanin production was reduced under the treatment of the miR-125b-5p mimic, and the expressions of melanin production-related proteins MITF, Tyr, Tyrp1, Tyrp2, and DCT were downregulated according to western blotting; meanwhile, overexpression of MITF reversed the above phenomenon, and the cellular melanin content increased. Compared with the treatment of miR-125b-5p mimic+NE-sh, the expressions of melanin-related proteins Tyr, Tyrp1, Tyrp2, MITF, and DCT were upregulated, and the suppression of MITF expression further enhanced the inhibitory influence of the miR-125b-5p mimic on melanogenesis, but the treatment of the miR-125b-5p mimic+sh MITF showed reduced cellular melanin content and downregulated expression of melanogenesis-related proteins MITF, Tyr, Tyrp1, Tyrp2, and DCT (Figures 6(b) and 6(c)).

## 4. Discussion

Vitiligo is an acquired pigmented skin disease resulting in a lack of pigment cells in the epidermis, which clinically manifests as white patches on the body that are symmetrically distributed, well-defined, and varying in size; it has a worldwide incidence of 0.5-1%. Segmental vitiligo (SV) and nonsegmental vitiligo (NSV) are the main types of vitiligo [11]. The development of vitiligo is a result of many factors. In the epidermis, vitiligo is caused by a variety of factors, but its main feature is damage to melanocytes between the hair follicles [12]. The unique biological function of melanocytes is melanin synthesis and plays a key role in the photoprotection of the skin; however, abnormal melanin reduction can have an obvious influence on the appearance and health of the individual. The study of melanin synthesis is of great importance for the treatment of vitiligo. Therefore, it is crucial to investigate new targets that can effectively promote melanin synthesis in melanocytes. miRNAs and some noncoding RNAs may play a key role in individual susceptibility to vitiligo [13]. In the present study, qRT–PCR assays revealed that miR-125b-5p expression was increased in the serum and tissues of vitiligo patients and was aberrantly expressed in the development of vitiligo. It has also been shown that altered gene expression is involved in melanogenic dysfunction, and the role of miRNAs in melanogenesis has been extensively studied. In melanoma, some miRNAs regulating melanogenesis have been found to play a key role [14]. miR-340 and miR-218 inhibit melanogenesis by negatively regulating MITF expression and thus play an inhibitory role in melanoma production [15, 16]. For example, miR-21a-5p, miR-25, and miR-27a-3p play a carcinogenic role in melanoma, and the levels are increased in metastatic melanoma [17, 18]. In this research, miR-125b-5p showed elevated expression in PIG3V compared to in PIG1 according to assay. The proliferative activity of melanocytes can be decreased by the overexpression of miR-125b-5p, blocking the cell cycle, increasing apoptosis, and decreasing the levels of melanogenesis-related proteins Tyr, Tyrp1, Tyrp2, and DCT, while reversing the inhibition of miR-125b-5p, such as shortening the G0/G1 and S phases, decreasing apoptosis, and upregulating melanogenesis-related protein expression. The results suggest that miR-125b-5p has a key role in melanogenesis in melanocytes.

miRNAs can regulate the level of related genes by binding to the 3′-UTR sequence of target gene mRNAs. MITF is a key developmental and differentiation program master transcriptional regulator that coordinates the melanocyte lineage [19, 20]. Several studies have shown a potential effect of MITF expression controlled by miRNAs and further regulate melanogenic enzymes at the mRNA level [21, 22]. For example, miR-508-3p overexpression led to decreased expression of MITF, Tyr, TyrP-2, and melanogenesis [23]. miR-137 also decreased MITF protein expression in transgenic mice [24]. In this study, the target binding between miR-125b-5p and MITF was predicted by a bioinformatics website, and the negative expression level of MITF was influenced by miR-125b-5p. Overexpression of miR-125b-5p resulted in weak proliferative viability, G0/G1 cell cycle arrest, and S phase arrest in melanocytes and increased apoptosis, while coexpression of MITF with miR-125b-5p significantly reversed the effect of miR-125b-5p overexpression. Moreover, knockdown of MITF increased the level of miR-125b-5p. The results indicate that miR-125b-5p affects the apoptosis and cycle of PIG1, proliferation, and PIG3V by targeting and regulating MITF. It is also a master regulator of three major pigmentation enzymes required for melanin synthesis: Tyr, TyrP1, and TyrP [25]. As a membrane-bound glycoprotein, Tyr plays a key role in melanin synthesis and is considered to be the rate-limiting enzyme for melanin synthesis, while TyrP1 and TyrP2, also known as dobutamine isomerase (DCT), act mainly in the later stages of melanin synthesis [26, 27]. In this study, after the level of miR-125b-5p was increased, the activity of Tyr in PIG1 and PIG2 cells was weakened, cellular melanin content was reduced, and the levels of MITF, Tyr, Tyrp1, Tyrp2, and DCT, which are proteins related to melanogenesis, were decreased. This phenomenon was obviously reversed after simultaneous overexpression of MITF, and if MITF was knocked down at the same time, the level of miR-125b-5 was enhanced, which affects melanogenesis of PIG1 and PIG3V through target regulation of MITF.

In summary, the level of miR-125b-5p was upregulated, and MITF was downregulated in vitiligo tissues and serum. miR-125b-5p regulates MITF expression, thereby affecting PIG1 and PIG3V cell proliferation, cell cycle progression, apoptosis, and melanogenesis, and it has a greater effect on PIG3V cells.

## Figures and Tables

**Table 1:** Primer sequence.

Genes	Primers	Sequence (5′-3′)
miR-125b-5p	forward	5′-TCCCTGAGACCCTAACTTGTGA-3′
	reverse	5′-AGTCTCAGGGTCCGAGGTATTC-3′

U6	forward	5′-CTCGCTTCGGCAGCACA-3′
	reverse	5′-AACGCTTCACGAATTTGCGT-3′

MITF	forward	5′-TCTGCCTGGTGCTGTACAAG-3′
	reverse	5′-CCAGGCCTTACCATCAGCAA-3′

GAPDH	forward	5′-AGTGATGGCATGGACTGTGG-3′
	reverse	5′-GATTTGGTCGTATTGGGCGC-3′

## Data Availability

The datasets used and/or analyzed during the current study are available from the corresponding author upon reasonable request.
